# Complete Genome Sequence of Bisphenol A-Degrading Bacterium *Sphingobium* sp. Strain A3, Isolated from Contaminated Soil

**DOI:** 10.1128/MRA.01088-20

**Published:** 2021-01-07

**Authors:** Ji Young Jung, Hye Kyeoung Kang, Dae Won Jeong, Hyun Mi Jin, Byung-Gon Ryu, Bok Yeon Jo, Eu Jin Chung, Sang-Soo Han

**Affiliations:** a Microbial Research Department, Nakdonggang National Institute of Biological Resources, Sangju-si, Gyeongsangbuk-do, South Korea; Loyola University Chicago

## Abstract

This study reports the complete genome sequence of bisphenol A-degrading bacterium *Sphingobium* sp. strain A3, which was isolated from a contaminated soil sample from the site of a factory fire in South Korea. The genome consists of a 6.53-Mbp chromosome and eight plasmid contigs (532,947 bp), with 6,406 protein-coding sequences and a GC content of 63.82%.

## ANNOUNCEMENT

Bisphenol A (BPA) is used in plastic bottles and food packaging (1, 2). BPA mimics the structure and function of the hormone estrogen, and it can interfere with normal bodily processes (3). Many studies have suggested that bacteria could provide a promising strategy for xenobiotic cleanup and bioremediation (4, 5). BPA-degrading bacteria, such as *Achromobacter* (6), *Pseudomonas* (7), *Bacillus* (8), and *Sphingomonas* (9), have been isolated. Although the isolation of novel bacteria led to proposed metabolic pathways of BPA degradation using intermediates detected during the degradation process, the genetic mechanisms of BPA degradation are not yet understood (10, 11).

The BPA-degrading bacterium *Sphingobium* sp. strain A3 was isolated from contaminated soil, and the whole genome was sequenced to understand its metabolic capacity and functional potential. Contaminated soil samples were collected at the site of a factory fire (35°18′55.1″N, 128°45′41.0″E) in the Gyeongsangnam-do province (South Korea). Enrichment cultures were conducted aerobically at 30°C for 2 weeks, with shaking, using contaminated soil in mineral salt medium (MSM) with 500 ppm BPA as the sole carbon source. The enrichment-cultured samples were diluted in phosphate-buffered saline (PBS) (pH 7.4). The dilutions were then spread on R2A agar plates (BD Difco, USA) and incubated at 30°C for 2 days to obtain a single colony. To determine the BPA-degrading activity using gas chromatography, following the protocols used in previous studies (12), each bacterial colony was seeded in 5 ml R2A medium, and the cells were inoculated into MSM with 300 ppm BPA at 30°C. After 24, 48, 72, and 92 h, the supernatants were obtained and analyzed to determine how much BPA had been removed. We screened the A3 bacterium, which had higher BPA-degrading activity among the single colonies obtained. This strain showed degradation rates of 51.6% ± 11.9%, 57.3% ± 8.1%, 86.9% ± 2.8%, and 96.9% ± 5.4% at 24, 48, 72, and 92 h, respectively.

Genomic DNA was isolated from the A3 strain using Maxwell 16 DNA purification kits (Promega, Madison, WI, USA). The genomic DNA was sequenced at Macrogen, Inc. (South Korea), using a combination of the PacBio RS II single-molecule real-time (SMRT) (13) sequencing platform, with a 20-kb SMRTbell template library, and the Illumina HiSeq X Ten sequencing platform (2 × 151 bp). For PacBio sequencing, genomic DNA (8 µg) was sheared to approximately 20 kb with a g-TUBE (Covaris) and purified using AMPurePB beads (Beckman Coulter), and the sequencing library was prepared using the SMRTbell template preparation kit v1.0 (PacBio). For Illumina sequencing, genomic DNA (100 ng) was sheared using an LE220 focused ultrasonicator (Covaris) and the sequencing library (350-bp insert size) was generated using a TruSeq Nano DNA library preparation kit. A total of 94,869 PacBio subreads (mean subread length, 6,496 bp; *N*_50_, 10,298 bp) were generated and used for preassembly and *de novo* assembly using FALCON-integrate software v2.1.4. A total of 12,165,610 raw Illumina paired-end reads (1.83 Gbp) were generated, and 5,664,022 clean reads (0.85 Gbp), in which ≥90% of the bases in each read had a Phred score of ≥30, were used for error correction with Pilon v1.21 for the final genome assembly. UGENE v1.32.0 was used to construct a self-dotplot to check the circularity of contigs. Overlapping ends were trimmed out. The genome was then annotated using NCBI Prokaryotic Genome Annotation Pipeline (PGAP) v4.12 (14). Default parameters were used for all software unless otherwise specified.

The final genome, assembly, and annotation statistics are shown in Table 1. The A3 genome was closely related to two strains, Sphingobium yanoikuyae ATCC 51230 (GenBank accession number NZ_CP023741.1) and Sphingobium scionense DSM 19371 (GenBank accession number JACIEU000000000.1), based on 16S rRNA sequence identity (100% and 99.1%, respectively), average nucleotide identity (95.79% and 92.66%, respectively), and digital DNA-DNA hybridization (68.0% and 50.6%, respectively) (15, 16).

**TABLE 1:** Summary of assembly and annotation statistics for *Sphingobium* sp. strain A3

Genetic element	Size (bp)	GC content (%)	No. of coding sequences	No. of rRNAs	No. of tRNAs	GenBank accession no.
Chromosome	6,531,504	63.92	6,176	12	73	CP060122
pSYA3-1	75,616	63.10	87	0	0	CP060124
pSYA3-2	71,501	59.74	72	0	0	CP060125
pSYA3-3	6,907	59.33	9	0	0	CP060130
Contig 5	232,139	63.48	247	0	1	CP060123
Contig 6	54,690	63.22	53	0	0	CP060126
Contig 7	54,171	61.40	61	0	0	CP060127
Contig 8	29,183	63.76	25	0	0	CP060128
Contig 9	8,740	62.95	8	0	0	CP060129
Total or average^*a*^	7,064,451	63.82	6,738	12	74	

a An average value for GC content (%) is indicated.

Although there is little information available on the enzymes and genes that are involved in the BPA degradation pathway, a few studies suggest that cytochrome P450 monooxygenase (17), laccase (18), lignin peroxidase (19), and manganese peroxidase (20) can degrade BPA. In the A3 genome, 10 P450 cytochromes were found in the chromosome and plasmid, and they showed high levels of similarity to previously reported P450 cytochromes of *Sphingobium* sp. strain YL23 (10, 21). Further in-depth biochemical and genomic analyses are needed to better understand BPA degradation pathways.

### Data availability.

The genome sequences and raw sequencing reads for the A3 strain were deposited under GenBank accession numbers CP060122, CP060123, CP060124, CP060125, CP060126, CP060127, CP060128, CP060129, and CP060130, BioProject accession number PRJNA649365, BioSample accession number SAMN15665156, and SRA accession numbers SRR12349700 and SRR12349701.

## References

[1] Baker ME, Chandsawangbhuwana C. 2012. 3D models of MBP, a biologically active metabolite of bisphenol A, in human estrogen receptor α and estrogen receptor β. PLoS One 7:e46078. doi:10.1371/journal.pone.0046078. 23056236 PMC3464279

[2] Umar M, Roddick F, Fan L, Aziz HA. 2013. Application of ozone for the removal of bisphenol A from water and wastewater: a review. Chemosphere 90:2197–2207. doi:10.1016/j.chemosphere.2012.09.090. 23153776

[3] Gao H, Yang B-J, Li N, Feng L-M, Shi X-Y, Zhao W-H, Liu S-J. 2015. Bisphenol A and hormone-associated cancers: current progress and perspectives. Medicine (Baltimore) 94:e211. doi:10.1097/MD.0000000000000211. 25569640 PMC4602822

[4] Ławniczak Ł, Woźniak-Karczewska M, Loibner AP, Heipieper HJ, Chrzanowski Ł. 2020. Microbial degradation of hydrocarbons: basic principles for bioremediation: a review. Molecules 25:856. doi:10.3390/molecules25040856. PMC7070569 32075198

[5] Singh K, Chandra S. 2014. Treatment of petroleum hydrocarbon polluted environment through bioremediation: a review. Pak J Biol Sci 17:1–8. doi:10.3923/pjbs.2014.1.8. 24783772

[6] Zhang C, Zeng G, Yuan L, Yu J, Li J, Huang G, Xi B, Liu H. 2007. Aerobic degradation of bisphenol A by *Achromobacter xylosoxidans* strain B-16 isolated from compost leachate of municipal solid waste. Chemosphere 68:181–190. doi:10.1016/j.chemosphere.2006.12.012. 17291567

[7] Eltoukhy A, Jia Y, Nahurira R, Abo-Kadoum MA, Khokhar I, Wang J, Yan Y. 2020. Biodegradation of endocrine disruptor bisphenol A by *Pseudomonas putida* strain YC-AE1 isolated from polluted soil, Guangdong, China. BMC Microbiol 20:11. doi:10.1186/s12866-020-1699-9. 31931706 PMC6958771

[8] Suyamud B, Inthorn D, Panyapinyopol B, Thiravetyan P. 2018. Biodegradation of bisphenol A by a newly isolated *Bacillus megaterium* strain ISO-2 from a polycarbonate industrial wastewater. Water Air Soil Pollut 229:348. doi:10.1007/s11270-018-3983-y.

[9] Fujiwara H, Soda S, Fujita M, Ike M. 2016. Kinetics of bisphenol A degradation by *Sphingomonas paucimobilis* FJ-4. J Biosci Bioeng 122:341–344. doi:10.1016/j.jbiosc.2016.02.015. 27038671

[10] Hu A, Lv M, Yu C-P. 2013. Draft genome sequence of the bisphenol A-degrading bacterium *Sphingobium* sp. strain YL23. Genome Announc 1:e00549-13. doi:10.1128/genomeA.00549-13. 23908290 PMC3731844

[11] Im J, Löffler FE. 2016. Fate of bisphenol A in terrestrial and aquatic environments. Environ Sci Technol 50:8403–8416. doi:10.1021/acs.est.6b00877. 27401879

[12] Vijayalakshmi V, Senthilkumar P, Mophin-Kani K, Sivamani S, Sivarajasekar N, Vasantharaj S. 2018. Bio-degradation of bisphenol A by *Pseudomonas aeruginosa* PAb1 isolated from effluent of thermal paper industry: kinetic modeling and process optimization. J Radiat Res Appl Sci 11:56–65. doi:10.1016/j.jrras.2017.08.003.

[13] Chin C-S, Alexander DH, Marks P, Klammer AA, Drake J, Heiner C, Clum A, Copeland A, Huddleston J, Eichler EE, Turner SW, Korlach J. 2013. Nonhybrid, finished microbial genome assemblies from long-read SMRT sequencing data. Nat Methods 10:563–569. doi:10.1038/nmeth.2474. 23644548

[14] Tatusova T, DiCuccio M, Badretdin A, Chetvernin V, Nawrocki EP, Zaslavsky L, Lomsadze A, Pruitt KD, Borodovsky M, Ostell J. 2016. NCBI Prokaryotic Genome Annotation Pipeline. Nucleic Acids Res 44:6614–6624. doi:10.1093/nar/gkw569. 27342282 PMC5001611

[15] Yoon SH, Ha SM, Lim J, Kwon S, Chun J. 2017. A large-scale evaluation of algorithms to calculate average nucleotide identity. Antonie Van Leeuwenhoek 110:1281–1286. doi:10.1007/s10482-017-0844-4. 28204908

[16] Meier-Kolthoff JP, Göker M. 2019. TYGS is an automated high-throughput platform for state-of-the-art genome-based taxonomy. Nat Commun 10:2182. doi:10.1038/s41467-019-10210-3. 31097708 PMC6522516

[17] Jia Y, Eltoukhy A, Wang J, Li X, Hlaing TS, Aung MM, Nwe MT, Lamraoui I, Yan Y. 2020. Biodegradation of bisphenol A by *Sphingobium* sp. YC-JY1 and the essential role of cytochrome P450 monooxygenase. Int J Mol Sci 21:3588. doi:10.3390/ijms21103588. PMC7278973 32438730

[18] Daâssi D, Prieto A, Zouari-Mechichi H, Martínez MJ, Nasri M, Mechichi T. 2016. Degradation of bisphenol A by different fungal laccases and identification of its degradation products. Int Biodeterior Biodegradation 110:181–188. doi:10.1016/j.ibiod.2016.03.017.

[19] Gassara F, Brar SK, Verma M, Tyagi RD. 2013. Bisphenol A degradation in water by ligninolytic enzymes. Chemosphere 92:1356–1360. doi:10.1016/j.chemosphere.2013.02.071. 23668961

[20] Hirano T, Honda Y, Watanabe T, Kuwahara M. 2000. Degradation of bisphenol A by the lignin-degrading enzyme, manganese peroxidase, produced by the white-rot basidiomycete, *Pleurotus ostreatus*. Biosci Biotechnol Biochem 64:1958–1962. doi:10.1271/bbb.64.1958. 11055402

[21] Sasaki M, Tsuchido T, Matsumura Y. 2008. Molecular cloning and characterization of cytochrome P450 and ferredoxin genes involved in bisphenol A degradation in *Sphingomonas bisphenolicum* strain AO1. J Appl Microbiol 105:1158–1169. doi:10.1111/j.1365-2672.2008.03843.x. 18492046

